# Case report: Varicella associated neuropsychiatric syndrome (VANS) in two pediatric cases

**DOI:** 10.1016/j.bbih.2023.100602

**Published:** 2023-02-11

**Authors:** Devika Dahiya, Claudia Marques Matos, Ming Lim, Ines Madureira, Sofia Duarte, Susan Byrne, Thomas Rossor

**Affiliations:** a The School of Medicine and Department of Paediatrics, Royal College of Surgeons in Ireland, Department of Paediatric Neurology, Dublin 2, Ireland; b Pediatric Neurology Department, Hospital Dona Estefânia, Centro Hospitalar Universitário Lisboa Central, Lisboa, Portugal; c Children's Neurosciences, Evelina London Children's Hospital at Guy's and St Thomas' NHS Foundation Trust, King's Health Partners Academic Health Science Centre, London, UK; d Department Women and Childrens Health, School of Life Course Sciences (SoLCS), Kings College London, UK; e Pediatric Rheumatology Unit, Hospital Dona Estefânia, Centro Hospitalar Universitário Lisboa Central, Lisboa, Portugal; f Department of Paediatric Neurology, Children's Health Ireland at Crumlin, Dublin 12, Ireland; g FutureNeuro, Royal College of Surgeons in Ireland, Dublin 2, Ireland

**Keywords:** Pediatric varicella zoster virus, Neuropsychiatric syndrome, Molecular mimicry, Cross reactivity, Auto-immune encephalitis, ADHD - Attention Deficit Hyperactivity Disorder, CNS – Central Nervous System, CSF – Cerebral Spinal Fluid, IVIG – Intravenous Immunoglobulin, NG – Nasogastric, NMDA - N-methyl-D-aspartate, PANDAS - Pediatric Autoimmune Neuropsychiatric Disorder Associated with Streptococcus), PANS - Pediatric Acute onset Neuropsychiatric Syndrome, PET- FDG - positron emission tomography - fluorodeoxyglucose, VZV - Varicella Zoster Virus

## Abstract

**Background:**

Viral or bacterial infections can trigger auto-immune inflammatory reactions and conditions in children. Self-reactivity arises due to similarities in molecular structures between pathogenic microorganisms and regular body structures with consequent immune-cross reactions. Reactivation of latent Varicella Zoster Virus (VZV) infections can cause neurological sequalae, including cerebellitis, post-herpetic neuralgias, meningo/encephalitis, vasculopathy and myelopathy. We propose a syndrome caused by auto-immune reactivity triggered by molecular mimicry between VZV and the brain, culminating in a post-infectious psychiatric syndrome with childhood VZV infections.

**Case presentation:**

Two individuals, a 6-year-old male and 10-year-old female developed a neuro-psychiatric syndrome 3–6 weeks following a confirmed VZV infection with intrathecal oligoclonal bands. The 6-year-old male presented with a myasthenic syndrome, behavior deterioration and regression in school, he was poorly responsive to IVIG and risperidone, however had a pronounced response to steroid treatment. The 10-year-old female presented with marked insomnia, agitation, and behavioral regression as well as mild bradykinesia. A trial of neuroleptics and sedatives resulted in a mild unsustained reduction in psychomotor agitation and IVIG was also unsuccessful, however the patient was very responsive to steroid therapy.

**Conclusion:**

Psychiatric syndromes with evidence of intrathecal inflammation temporally related to VZV infections that are responsive to immune modulation have not been described before. Here we report two cases demonstrating neuro-psychiatric symptoms following VZV infection, with evidence of persistent CNS inflammation following the resolution of infection, and response to immune modulation.

## Highlights

• Viral or bacterial infections can trigger auto-immune conditions in children.
• Similar molecular structures in microorganisms and the body triggers self-reactivity.
• Neuro-psychiatric symptoms following varicella infection have not been described.
• Behavioral changes following infection were responsive to immune modulation.

## 1 Introduction

Auto-immune inflammatory conditions can be precipitated by viral or bacterial infections in children (Getts et al., 2013; Smatti et al., 2019). Similar molecular structures between pathogenic microorganisms and native molecules can result in immune cross-reaction and a self-reactive phenotype with the generation of auto-antibodies that target endogenous structures (Tanaka et al., 2020). Isolated psychiatric syndromes without specific neurological involvement triggered by autoimmunity are very rare, however some reported series of auto-immune encephalitis (AE) have only presented with neuropsychiatric issues, in particular a cohort of young people with NMDA receptor antibodies (Endres et al., 2020). In addition, there has been recent description of autoimmune neuropsychiatric disorders associated with streptococcal infections. These are characterised by sudden onset obsessive compulsive behaviour or food restriction accompanied by neuropsychiatric symptoms including behavioural regression, emotional lability, anxiety and motor and sensory deficits, but without associated markers of intrathecal inflammation or a known antibody (Wilbur et al., 2019).

At the current time treatment for streptococcal infection associated autoimmune neuropsychiatric syndromes differs from the AEs ((BPNA) TBPNA, 2021).

Varicella Zoster Virus (VZV) infections are known to cause neurological sequalae, specifically via reactivation of latent virus and zoster infections (Gilden et al., 2013), including cerebellitis, post-herpetic neuralgias, meningo/encephalitis, vasculopathy and myelopathy (Gilden et al., 2013; Nagel and Gilden, 2014). We propose a syndrome caused by auto-immune reactivity triggered by molecular mimicry between VZV and the brain, culminating in a post-infectious psychiatric syndrome with childhood VZV infections. Psychiatric syndromes with evidence of intrathecal inflammation temporally related to VZV infections that are responsive to immune modulation have not been described before. Here we report two cases demonstrating neuro-psychiatric symptoms following VZV infection, with evidence of persistent CNS inflammation following the resolution of infection, and response to immune modulation.

## 2 Case descriptions

**Fig. 1:**
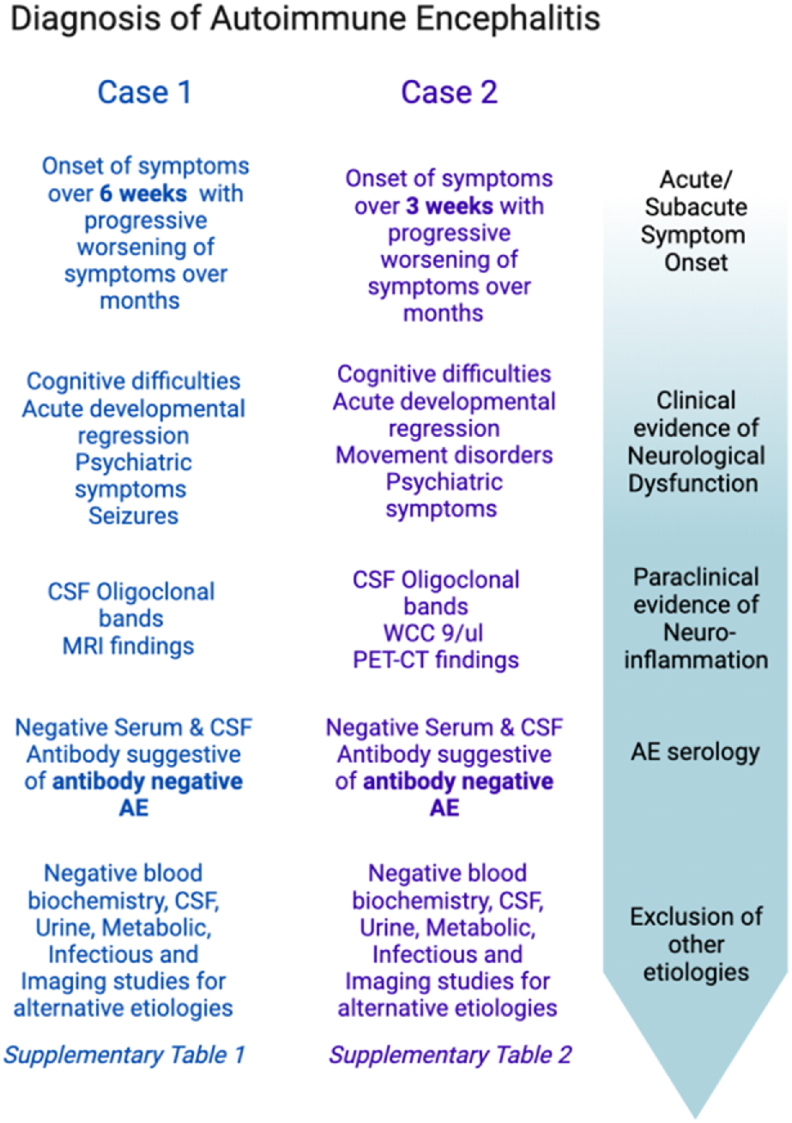

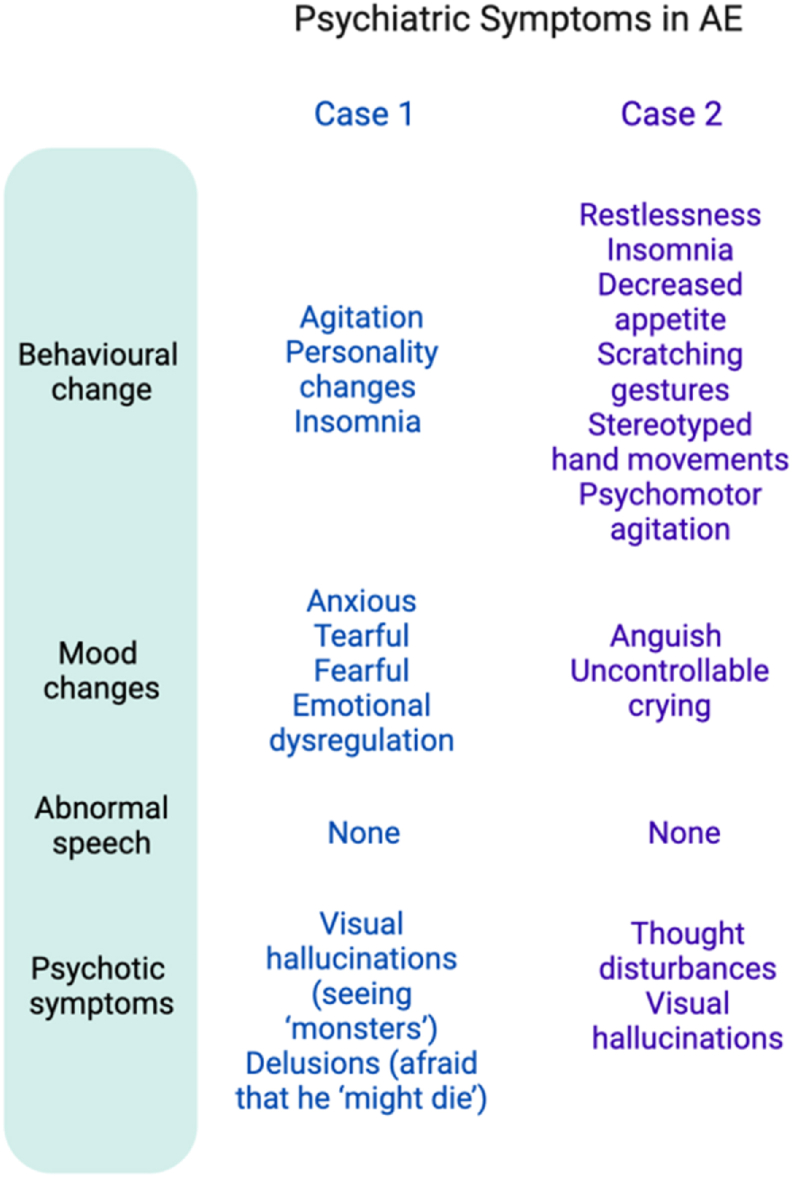
**(A)** Cellucci criteria (Cellucci et al., 2020) to diagnose autoimmune encephalitis in the paediatric patient as applied to both cases. **(B)** Psychiatric symptoms in the reported cases of paediatric autoimmune encephalitis characterised as per Rosello et al.’s framework (Nagel and Gilden, 2014).

Both case 1 and case 2 met the diagnostic criteria of autoimmune etiology of pediatric encephalitis as described by Cellucci et al. (2020) (see Fig. 1A). Psychiatric symptoms were characterized using a framework reported by Rosello et al. (2022) which characterizes symptomatology in autoimmune encephalitis (see Fig. 1B).

### 2.1 Case 1

A 6-year-old boy was referred to pediatric neurology ten days after VZV infection with new onset ptosis and bulbar weakness requiring NG feeding. In addition, he was not behaving in his usual manner and had one brief seizure associated with pyrexia. Premorbidly, he was a bright boy with normal developmental and educational attainment. There was no family history of note. His clinical and neurophysiology assessments were in keeping with myasthenia. Myasthenia-associated antibodies were negative, and he responded to pyridostigmine. He had 21-days of acyclovir therapy. Six weeks later he was reviewed in clinic and parents noted a marked change in behavior. Behavioral management techniques were employed, and he partially responded. However, over the course of the next 6 months his behavior markedly deteriorated. When he was seen in clinic 12-months after his initial varicella infection, his myasthenic syndrome had abated and he was taken off pyridostigmine, however he was noted to be extremely anxious, tearful, and fearful, and with emotional dysregulation. In addition, he complained of seeing ‘monsters’ and was afraid that he ‘might die’. He could no longer go to school regularly and his handwriting had been noted to deteriorate markedly (see Supplementary Fig. 1). He had developed separation anxiety that was so extreme that his mother could no longer attend work.

Fourteen months after initial presentation, he represented with emergency of a few vesicular lesions which crusted over. He was treated with one week of oral acyclovir for a presumed VZV reinfection, but no molecular testing for VZV infection was carried out. The parents associated this infection was a marked deterioration in behaviour.

Extensive work-up was negative (see Supplementary Table 1) except for detection of intrathecal synthesis of IgG with positive oligoclonal bands on two occasions. In addition, he had a cerebellar lesion that enhanced with gadolinium. He was given a trial of IVIG (2g/kg delivered over two days) with limited benefit. He was started on risperidone at 0.25mg once daily which did lessen symptoms somewhat, and normalized behaviour, but he remained unable to attend school. A decision was made to give a pulse of steroids. He had a remarkable response to steroids with resolution of the majority of symptoms, return to mainstream school, and improvement in his handwriting. A few days before the next pulse of steroid was required parents would note a recurrence of the symptoms. After 12- months of pulsed dexamethasone therapy (6mg/kg in 2 divided doses per day for 3 days) he was commenced on Mycophenolate mofetil and steroids subsequently discontinued. Four years on from presentation he has improved but is not at his pre-morbid baseline; on-going issues with attention and mood fluctuation. He takes Aripiprazole and Guanfacine.

### 2.2 Case 2

A 10-year-old girl was admitted to the paediatric neurology/psychiatry ward with a 6-month-long history of progressive behavioural disturbances, characterized by marked restlessness and insomnia evolving with anguish, uncontrollable crying, psychomotor agitation, thought disturbances and visual hallucinations. Three weeks prior to the onset of the behavioural disturbance she had chickenpox. At admittance, she was oriented in time and space, reported seeing frightening images of accidents with blood. She exhibited frequent scratching gestures and complained of itching and reduced appetite and insomnia. On examination her eye-contact and attention were fleeting, and she had stereotyped hand movements. In addition, there was slight bradykinesia with normal tone. There were no focal neurological features. She has a history of mild intellectual disability, fine motor skills difficulties, learning difficulties and attention deficit hyperactivity disorder (ADHD), treated for one year with Methylphenidate 20mg prior to onset of her current difficulties. She had an episode of Enterovirus meningitis at 4 years of age. There was a first-degree relative with depression and another with intellectual disability.

Extensive etiological work-up (see Supplementary Table 2) was positive only for the detection of intrathecal synthesis of IgG with positive oligoclonal bands (profile 2) on 2 different occasions, 12 months apart. Symptomatic treatment was tried with neuroleptics (risperidone, haloperidol, aripiprazole, levopromazine, ciamemazine), lorazepam, clonazepam, valproate, clonidine and atomoxetine. This ameliorated symptoms of anxiety, insomnia and decreased appetite and enabled some visual contact and some drawing and writing activities but the wandering, restlessness and soliloquy remained. Three cycles of IVIG (1mg/kg/day for 2 days, every 3 weeks) were administered, resulting in only mild, unsustained, reduction in psychomotor agitation. A second trial of IVIG (1 cycle) was used following a flare-up of symptoms, with food refusal and significant weight loss, with no benefit.

Subsequently a brain PET-CT FDG scan revealed discrete parietal and right occipital hypometabolism suggestive of encephalitis and after multidisciplinary discussion, a course of IV methylprednisolone was administered (30mg/kg/day for 5 days), which resulted in reduced wandering, maintained eye contact and conversation and regularization of sleep and appetite with normalisation of body weight. Moreover, she was able to resume school. Owing to the remarkable response to immunotherapy, mofetil mycophenolate was added (target dose 750mg bid) to monthly 3-day-long pulses of 30mg/kg/day methylprednisolone and a 6-month-long deescalating scheme of oral prednisolone (starting with 60mg/day). 22 months after the first course of methylprednisolone and mycophenolate mofetil, she has improved and is back in school. 17 months after the first steroid course there was another episode of behaviour aggravation, insomnia and agitation. She was given another pulse of 3 days methylprednisolone 30mg/kg/day followed by a 1-month course of oral prednisolone 1mg/kg/day with taper which was stopped due to an absence of response. A repeat brain MRI at this time was normal. 2 months following this behavioural aggravation, the patient was better and we are convinced that this episode is not in relation with the disease. She currently takes Mycophenolate Mofetil (750 + 1000mg).

## 3 Discussion

Here we present two children who developed marked behavioral change persisting for months and resulting in inability to attend school following infection with varicella zoster. Both had unpaired oligoclonal bands in the CSF which persisted many months beyond VZV infection, and both patients responded to treatment with steroids. We suspect an immune mediated pathology in these instances, whereby an acute VZV infection triggers auto-immunity through molecular mimicry between the virus and the brain. VZV has been identified as a trigger for other types of neurological disorders, including multiple sclerosis (Kattimani and Veerappa, 2018) and Guillain-Barre syndrome (Lehmann and Hartung, 2010), the mechanism being linked to molecular mimicry. It is feasible that antigenic similarities noted between VZV, and the brain results in cross-reactivity, culminating in a post-infectious psychiatric syndrome with childhood VZV infections. There are documented cases of auto-reactive anti-NMDA receptor antibody production and consequent NMDA receptor encephalitis following VZV infection all of which meet the Cellucci criteria for AE (Fatma et al., 2022; Schäbitz et al., 2014). In the cases reported by Schäbitz et al. and Fatma et al. both patients demonstrated significant improvement to combined treatment with an antiviral and immunomodulatory IV steroids, with minimal symptoms persisting at follow up, drawing similarities to the two cases presented here (Fatma et al., 2022). In another report by Prakash et al., a 50 year old woman developed a complex neuro-symptom onset including breath holding spells, her CSF PCR was positive for VZV and anti-NMDA receptor antibody, meeting the Cellucci criteria for AE (Prakash et al., 2019). In this case the patient only received anti-viral treatment which normalized vital signs and decreased agitation however the patient did not reach neurological baseline by discharge. In another case a patient with VZV developed anti-Gly receptor antibody positive progressive encephalomyelitis with rigidity and myoclonus (PERM) (Yuan et al., 2022). Treatment with IV steroids and immunoglobulins reduced severity and frequency of spasms in this patient. These cases demonstrate auto-immune cross-reactivity between VZV and native central nervous system structures that can manifest in neuro-psychiatric symptoms.

In the case of the 6-year-old male, one of the presenting symptoms was antibody negative myasthenic syndrome responsive to pyridostigmine. Myasthenic symptoms were absent in the 10-year-old female. While myasthenic syndromes have not been reported in conjunction with VZV infections, Herpes Simplex Virus (HSV) infections, which are also a part of the Herpesvirus family, have been reported to result in myasthenia gravis secondary to molecular mimicry between herpes simplex virus glycoprotein D and the human acetylcholine receptor (Eddy et al., 1999; Schwimmbeck et al., 1989). In addition, CNS HSV infection has been associated with a secondary NMDA encephalitic syndrome (Leypoldt et al., 2013), presumably due to a break-down in the blood brain barrier and epitope exposure.

We hypothesize that the VZV infection triggered the production of antibodies that cross react with components of the motor-end plate (patient 1) as well as endogenous neuroproteins (patients 1 and 2). Both cases had tested positive for intrathecal bands following infection, which was temporally associated with the neuropsychiatric symptoms. Interestingly, while neither of the patients had a sustained response to IVIG, both cases demonstrated a robust response and symptom resolution with steroids. Steroid responsiveness with positive CSF band testing provides evidence to support the theory that the underlying mechanism of the neuro-psychiatric syndrome seen is due to cross-reactivity of anti-VZV antibodies and the brain.

Auto-immune encephalitis in a pediatric population very often presents with pronounced neuro-psychiatric symptoms (Shekunov et al., 2020). PANS (pediatric acute onset neuropsychiatric syndrome) is a separate entity characterized by sudden onset neuro-psychiatric symptoms, typically including obsessive compulsive and food-restrictive behaviors, theorized to be caused by infections, metabolic and endocrine disorders, or neuro-inflammation ((BPNA) TBPNA, 2021; Swedo et al., 2017). Post-streptococcal autoimmunity with obsessive compulsive and anxiety behaviors amongst other psychiatric symptoms are classified as PANDAS (pediatric autoimmune neuropsychiatric disorder associated with streptococcus). PANS and PANDAS have not had oligoclonal band positivity demonstrated to date, which indicate active neuroinflammation, and the exact trigger is typically unknown, which is why we propose that these post-varicella syndromes reported require a separate classification.

Psychiatric syndromes with evidence of intrathecal inflammation temporally related to VZV infections that are responsive to immune modulation have not been described before. We propose the term Varicella Associated Neuropsychiatric Syndrome (VANS) to classify these syndromes.

We highlight the importance of careful monitoring of children with varicella with close attention to changes in their mental state and new onset psychiatric symptoms, by their parents, carers and by doctors. Urgent reporting in cases of deterioration of mental status during the course of the infection may enable rapid diagnosis of VANS and early treatment may optimise disease course and outcomes. In addition, closer monitoring and early reporting will further our understanding of the spectrum of symptoms and signs of VANS. We propose that pediatric patients with confirmed Varicella Zoster Virus infections meeting the Celluci criteria for Autoimmune Encephalitis and new onset psychiatric symptoms (≥2 categories from Rosello's framework) temporally related to the infection meet the diagnostic criteria for VANS. Additional single reports and larger series of cases that meet these criteria are required to corroborate these findings and further establish the spectrum of symptoms and signs seen in varicella associated neuropsychiatric syndrome in paediatric patients.

## Declaration of competing interest

M.J. Lim receives research grants from Action Medical Research, the DES society, the GOSH charity, the National Institute for Health Research, the MS Society, and the SPARKS charity; receive research support grants from the London Clinical Research Network and the Evelina Appeal, has received consultation fees from CSL Behring, Novartis and Octapharma, has received travel grants from Merck Serono, and was awarded educational grants to organise meetings by Novartis, Biogen Idec, Merck Serono, and Bayer.

The authors declare that they have no known competing financial interests or personal relationships that could have appeared to influence the work reported in this paper.

## Data Availability

Data will be made available on request.
