# Supplementary material for: Case report: Varicella associated neuropsychiatric syndrome (VANS) in two pediatric cases
Source: Brain Behav Immun Health. 2023 Feb 11;28:100602. doi: 10.1016/j.bbih.2023.100602 (PMC9969201; doi:10.1016/j.bbih.2023.100602)
Supplement: Multimedia component 1 [file mmc1.zip › mmc1.DOCX]

**Case Report: Varicella Associated Neuropsychiatric Syndrome (VANS) in two pediatric cases - Supplementary Data**

**Table 1 – Diagnostic Workup Case 1**

| **Normal studies** | **Abnormal studies** |
| --- | --- |
| **Blood tests**  Acetylcholine receptor antibodies  Acetylcholine receptor cluster antibodies  Muscle kinase antibodies  Muscle kinase cluster antibodies  LRP4 antibodies  Antinuclear antibodies  Anti ri, Hu, Yo  Basal ganglia antibodies  NMDA receptor antibodies  Immunoglobulins; IG1-4  Vacuolated lymphocytes  Ammonia, acyl carnitines, amino acids, lactate  Tissue transglutaminase  Copper, caeruloplasmin, ESR,  Bone profile, Vitamin D, B12  Thyroid function testing  ACTH, cortisol, thyroid, FSH, LH, testosterone  EBV antibodies  CMV antibodies  Borrelia burgdoferi antibodies  T spot  **CSF**  CSF WCC <1, RCC 17  CSF protein, lactate  Pyridoxal phosphate  Amino acids (serine, threonine, glycine, amino acids)  Herpes simplex virus DNA not detected  Varicella zoster virus DNA not detected  CSF measles, rubella and herpes simplex IgG  Total IgG ratios suggest intrathecal antibody production (on 2 occasions), no specific intrathecal IgG detected  **Urine biochemistry**  Homovanillic acid  5-hydroxyindoleacetic acid  Urinary metanephrines  Urinary cortisol  **Imaging**  MRI chest, abdomen, pelvis  **EEG**  **Further imaging**  Whole body MRI  PET FDG half body | **CSF 1 and 2**  CSF oligoclonal bands raised (serum normal)  **MRI brain presentation** reported as mild cerebellitis  **MRI Brain at 12 months**  Small focus of signal change in the left peridentate cerebellum. This is of uncertain significance and could be inflammatory or an incidental hamartoma.    **Nerve conduction studies/ EMG** within normal limits, mild abnormalities on repetitive stimulation with increased decremental response  **Infection bloods**  Varicella IgG positive |

**Figure 1 – Handwriting Samples Case 1**

**
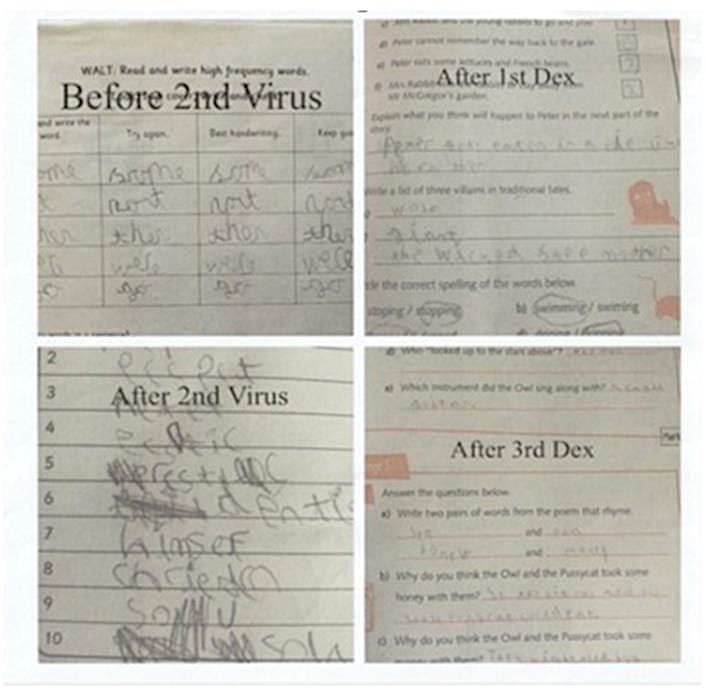
**

**Table 2 – Diagnostic Workup Case 2**

| **Normal studies** | **Abnormal studies** |
| --- | --- |
| **Brain MRI** (at first admission and at 24 months)  **EEG** (adequate for age, in sleep there is a very slight paroxysmal activity in the rolandic region)  **Blood tests**  Complete blood count, ESR  Kidney function  Complete liver function  Iron metabolism  Vitamin B12, homocysteine and folate levels  Lipid profile  Protein electrophoresis  Lactate dehydrogenase  Creatine kinase  Anti-thyroid antibodies  ANA  Anti-gliadin and anti-transglutaminase (IgA and IgG)  Anti-neuronal surface antibodies   - NMDAR in serum and CSF, AMPA 1 and 2, GABA-B receptor, LGI1 in serum - NMDAR, AMPA, GABA-A and GABA-B receptors, mGluR1, mGluR5, LGI1, Caspr2, DPPX, Neuroxin3 and Iglon5^[[1]](#endnote-1)^   Others (anti-MOG, anti-gangliosides and onconeuronal antibodies in serum)  Serologies in serum (*M. pneumoniae, B. burgdorferi, C.burnetti, Rickettsia, Bartonella,* Arboviruses and HIV1/2  Bengal Rose reaction and blood cultures (*Brucella*)  ASO and anti-DNase B titers negative  Stool cultures (*Shigella, Salmonella* and *Campylobacter*, parasites and *Clostridium dificille)*  Thyroid function  Ammonia  Lactate  Serum and urinary copper and ceruloplasmin  **CSF analysis**  Normal glucose, protein, LDH and lactate  Negative culture  Herpes simplex virus DNA not detected  Varicella zoster virus DNA not detected  Molecular biology (HSV 1/2, VZV, HHV 6, 7 and 8, EBV, CMV and Enterovirus)  **Urine biochemistry**  Urinary catecholamines  Prolactin – slightly elevated (37.62-64.14 ng/mL but normal at discharge 14.99 ng/mL *reference values* 4.20-23.04)  **Imaging**  Abdominal and pelvic ultrasound  **Genetic studies**  Next-Generation Sequencing gene panel of 1174 genes of syndromic intellectual disability | **1^st^ CSF analysis**  9 cells/uL; presence of oligoclonal bands (> 5) in CSF alone (profile 2); normal IgG index of 0.43  **2^nd^ CSF (at 12 months):**  2 cells/uL; presence of oligoclonal bands (> 5) in CSF alone (profile 2); normal IgG index of 0.73  **Brain PET/CT FDG scan**  Discrete cerebral hypometabolism (parietal and right occipital) in probable relationship with a clinical picture of encephalitis |

1. Serum and CSF analysed at the research lab of Professor Josep Dalmau in Barcelona through a comprehensive screening [↑](#endnote-ref-1)
